# Serotonergic modulation of post-synaptic inhibition and locomotor alternating pattern in the spinal cord

**DOI:** 10.3389/fncir.2014.00102

**Published:** 2014-08-28

**Authors:** Florian Gackière, Laurent Vinay

**Affiliations:** Institut de Neurosciences de la Timone, UMR 7289, CNRS, Aix Marseille UniversitéMarseille, France

**Keywords:** 5-HT2A receptor, 5-HT7 receptor, chloride homeostasis, KCC2 transporter, reciprocal inhibition

## Abstract

The central pattern generators (CPGs) for locomotion, located in the lumbar spinal cord, are functional at birth in the rat. Their maturation occurs during the last few days preceding birth, a period during which the first projections from the brainstem start to reach the lumbar enlargement of the spinal cord. Locomotor burst activity in the mature intact spinal cord alternates between flexor and extensor motoneurons through reciprocal inhibition and between left and right sides through commisural inhibitory interneurons. By contrast, all motor bursts are in phase in the fetus. The alternating pattern disappears after neonatal spinal cord transection which suppresses supraspinal influences upon the locomotor networks. This article will review the role of serotonin (5-HT), in particular 5-HT_2_ receptors, in shaping the alternating pattern. For instance, pharmacological activation of these receptors restores the left-right alternation after injury. Experiments aimed at either reducing the endogenous level of serotonin in the spinal cord or blocking the activation of 5-HT_2_ receptors. We then describe recent evidence that the action of 5-HT_2_ receptors is mediated, at least in part, through a modulation of chloride homeostasis. The postsynaptic action of GABA and glycine depends on the intracellular concentration of chloride ions which is regulated by a protein in the plasma membrane, the K^+^-Cl^−^ cotransporter (KCC2) extruding both K^+^ and Cl^−^ ions. Absence or reduction of KCC2 expression leads to a depolarizing action of GABA and glycine and a marked reduction in the strength of postsynaptic inhibition. This latter situation is observed early during development and in several pathological conditions, such as after spinal cord injury, thereby causing spasticity and chronic pain. It was recently shown that specific activation of 5-HT_2A_ receptors is able to up-regulate KCC2, restore endogenous inhibition and reduce spasticity.

## Introduction

It is well established that the basic rhythmic activity underlying locomotion is generated by interneuronal networks within the spinal cord called central pattern generators (CPGs; Grillner and Wallén, 1985). These are functional at birth in the rat as shown by experiments using *in vitro* spinal cord preparations isolated from neonates (Cazalets et al., 1992). In these preparations, the most effective pharmacological cocktails to induce fictive locomotion include serotonin (5-HT; Cazalets et al., 1992; Madriaga et al., 2004). There is considerable evidence that 5-HT plays a key role in locomotion. Chronic recordings from 5-HT neurons in awake cats demonstrated a correlation between single unit activity and locomotor activity (Veasey et al., 1995) suggesting that the 5-HT system facilitates motor output and concurrently inhibits sensory information processing (Jacobs and Fornal, 1993). Stimulation of a discrete population of 5-HT neurons in the parapyramidal region (PPR) of the medulla elicits locomotor-like activity in the neonatal rat isolated brain stem-spinal cord preparation (Liu and Jordan, 2005). Most locomotor-activated cells, as revealed by expression of the activity-dependent marker *c-fos*, co-localize with 5-HT_7_, 5-HT_2A_, and 5-HT_1A_ receptors (Noga et al., 2009). The intrinsic 5-HT system contributes significantly to locomotor pattern generation (Zhang and Grillner, 2000; Pearlstein et al., 2005, 2011). In addition, there is increasing evidence that recovery of locomotion after spinal cord injury (SCI) can be facilitated by systemic or intrathecal application of 5-HT or various 5-HT receptor agonists (Feraboli-Lohnherr et al., 1999; Kim et al., 2001; Antri et al., 2002, 2005; Landry et al., 2006), or transplantation of embryonic 5-HT neurons into the spinal cord caudal to the lesion (Feraboli-Lohnherr et al., 1997; Ribotta et al., 1998a,b, 2000; Kim et al., 1999; Sławińska et al., 2000, 2013; Majczyński et al., 2005).

Serotonin has a number of effects on the spinal cord, including the control of motoneuron and interneuron excitability and afferent transmission (Schmidt and Jordan, 2000; Abbinanti and Harris-Warrick, 2012; Abbinanti et al., 2012). The present review will focus on the contribution of 5-HT, with emphasis on 5-HT_2_ and 5-HT_7_ receptors, in shaping the alternating pattern, and on one of the mechanisms underlying this effect, strengthening of post-synaptic inhibition, by modulation of chloride homeostasis.

## Endogenous serotonin is important for the expression of a left-right alternating pattern

Pharmacological activation of the CPGs *in vitro* in newborn animals evokes a fictive locomotor pattern consisting of alternation of motor bursts between both the left and right sides of the lumbar spinal cord, and flexors and extensors on one side (Cazalets et al., 1992; Kiehn and Kjaerulff, 1996). On the embryonic day (E)16 (i.e., 5 days prior to birth), the same kind of experiments reveal a motor pattern with all bursts in phase (Iizuka et al., 1998; Nakayama et al., 2002). In rats, the transition from left-right synchrony to alternation occurs around E18 and is due to the maturation of inhibitory connections between the two sides, and a shift in GABA/glycine synaptic potentials from excitation to inhibition (Wu et al., 1992; see below).

These major changes in locomotor network operation occur shortly after the arrival in the lumbar enlargement of the first axons descending from the brainstem, suggesting that descending pathways may contribute to the maturation of spinal networks (Vinay et al., 2000, 2002). Serotonergic fibers start to arrive in the lumbar gray matter by E17 (Bregman, 1987; Rajaofetra et al., 1989). Projections arising from the raphe nuclei are among the earliest axons to reach the upper lumbar segments in the rat (Lakke, 1997). They are the source of almost all the 5-HT in the lumbar spinal cord in mammals (reviewed by Schmidt and Jordan, 2000).

A number of experiments support the conclusion that descending pathways, in particular 5-HT projections, play a role in the maturation and/or the operation of the lumbar CPG. Daily *in vivo* injections of p-chloro-phenylalanine (PCPA), a 5-HT synthesis inhibitor, starting the day of birth markedly reduce 5-HT immunoreactivity in the lumbar enlargement within 3–4 days (Pflieger et al., 2002). Depletion of endogenous 5-HT during early postnatal development induces an asymmetry of posture (Pflieger et al., 2002) and deficits in locomotion (Myoga et al., 1995), both of which indicate that the interlimb coordination is impaired. In addition, kittens or rats that have undergone a complete spinal cord transection at birth exhibit synchronous air stepping during the first postnatal week (Bradley and Smith, 1988a,b; Norreel et al., 2003). Quipazine, a 5-HT_2_ receptor agonist promotes alternating air stepping in intact neonatal rats (Brumley et al., 2012). The 5-HT_7_ receptors also appear to play an important role as the antagonist, SB-269970, applied directly to the spinal cord consistently disrupts locomotion in adult mice (Liu et al., 2009).

**Figure 1:**
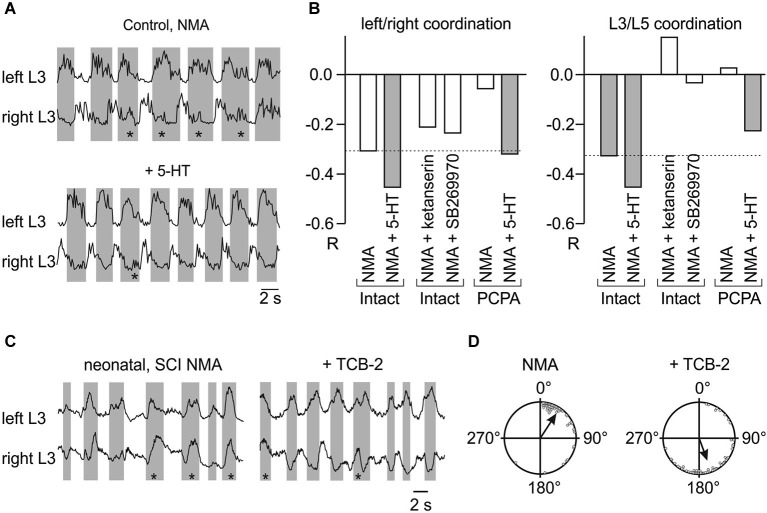
**5-HT enhances the NMA-induced fictive locomotor pattern in the isolated neonatal rat spinal cord. (A)** Activity recorded from the 3rd left and right lumbar ventral roots after rectification and integration. Bath application of NMA (18 μM) induced fictive locomotion characterized by left-right alternation. Addition of 5-HT (5 μM) stabilized these alternations. Note the lower occurrence of left-right concomitant bursting (asterisks). **(B)** Mean correlation coefficients (R) for left-right and L3/L5 relationships in various experimental conditions, after adding 5-HT, blocking 5-HT_2A_ receptors with ketanserin or 5-HT_7_ receptors with SB269970, or blocking 5-HT synthesis by PCPA. **(A)** and **(B)** are adapted from Pearlstein et al. (2005). **(C,D)** Activation of 5-HT_2A_ receptors *in vitro* restores the left-right alternating locomotor pattern 5 days after neonatal spinal cord transection. **(C)** Integrated recordings from the left and right L3 ventral roots at P5 in the presence of NMA (16 μM) alone (note the high occurrence of synchronous bursts, asterisks) or together with 5-HT_2A_ receptor agonist (4-bromo-3,6-dimethoxybenzocyclobuten-1-yl) methylamine hydrobromide (TCB-2): (0.1 μM). **(D)** Distribution of phase relationships between left and right ventral root bursts in NMA alone (Left) and NMA plus TCB-2 (Right) in all of the spinal animals (P5–P6; *n* = 6). **(C)** and **(D)** are adapted from Bos et al. (2013).

*In vitro* experiments showed that 5-HT, when added with *N*-methyl-d,l-aspartate (NMA) to neonatal rat isolated spinal cord preparations, strongly strengthens left/right and flexor/extensor alternation, an effect that is at least partly dependent on activation of 5-HT_2_ receptors (Figures 1A,B; Pearlstein et al., 2005). The NMA-induced motor pattern is strongly affected in PCPA-treated animals (Pearlstein et al., 2005). Both left/right and L3-L5 alternations are weak but recover after adding 5-HT (Figure 1B). A contribution of endogenous 5-HT is further supported by the observations that ketanserin (a 5-HT_2_ receptor antagonist, Figure 1B) or SB-269970 (a 5-HT_7_ receptor antagonist, Figure 1B) disorganizes the locomotor pattern (makes the cross-correlation coefficient less negative) induced by either NMA (Pearlstein et al., 2005; Liu et al., 2009; Jordan and Slawinska, 2011) or electrical stimulation of the brainstem (Liu and Jordan, 2005). Finally, in spinal cords isolated from 5-HT_7_ receptor knock-out mice, 5-HT produces either uncoordinated rhythmic activity or results in synchronous discharges of the ventral roots (Liu et al., 2009).

Together, these observations suggest that 5-HT_2_ and 5-HT_7_ receptors appear to mediate the effect of serotonin to enhance and stabilize both left-right and flexor-extensor alternation. Other 5-HT receptors, such as 5-HT_1A_, may have an opposite effect to reduce reciprocal inhibition as shown in *Xenopus laevis* (Wedderburn and Sillar, 1994; McDearmid et al., 1997).

## The strength of postsynaptic inhibition is reduced after SCI due to a dysregulation of chloride homeostasis

Ipsilateral co-contraction of flexors and extensors is commonly observed in SCI patients (Harkema, 2008). The strength of several well-characterized inhibitory mechanisms such as presynaptic (Katz, 1999), recurrent (Mazzocchio and Rossi, 1997) and reciprocal (Boorman et al., 1996) inhibition is reduced after SCI. A reciprocal facilitation, instead of reciprocal inhibition, may even appear (Crone et al., 2003). Similarly, crossed inhibition of contralateral motoneurons by group II muscle afferents in intact cats is inverted to crossed excitation in spinal cats (Aggelopoulos et al., 1996). The mechanisms responsible for the decrease in strength of postsynaptic inhibition were recently identified. Briefly, in healthy mature cells, activation of GABA_A_ and glycine receptors leads to chloride entry which causes membrane hyperpolarization. This occurs because the intracellular concentration of chloride ions ([Cl^−^]_i_) is maintained at low levels by the potassium-chloride co-transporters KCC2 that extrude chloride from the cell (Payne et al., 2003; Vinay and Jean-Xavier, 2008; Blaesse et al., 2009; Chamma et al., 2012). There is now abundant evidence that an increase in [Cl^−^]_i_, most often recorded/visualized as a depolarizing shift of the chloride equilibrium potential, reduces the strength of postsynaptic inhibition or may even switch it towards excitation or promote facilitation of concomitant excitatory inputs (van den Pol et al., 1996; Gao et al., 1998b; Gulledge and Stuart, 2003; Prescott et al., 2006; Jean-Xavier et al., 2007; Doyon et al., 2011).

The expression of KCC2 in the plasma membrane of lumbar motoneurons below the lesion is reduced after spinal cord injury, thereby causing a depolarizating shift in the chloride equilibrium potential (Boulenguez et al., 2010). Similar results were described in the superficial layers of the dorsal horn either after SCI (Cramer et al., 2008; Lu et al., 2008) or following peripheral nerve injury (Coull et al., 2003, 2005). These observations were shown to contribute to spasticity and chronic pain, respectively. To conclude, dysregulation of chloride homeostasis can account for the reduction in strength of postsynaptic inhibition or even a switch to facilitation after SCI.

## Serotonin enables restoration of coordinated locomotion after spinal cord injury

Following a neonatal spinal cord transection that disorganizes the left-right alternating pattern, left-right hindlimb alternation is restored after injecting (±)-2,5-dimethoxy-4-iodoamphetamine hydrochloride (DOI), a 5-HT_2_ receptor agonist (Norreel et al., 2003). In addition, however, sensory inputs from the moving limbs *in vivo* can also promote left-right alternations under certain circumstances. In kittens with spinal cord transection at birth, alternation is more pronounced during treadmill stepping (~40% of alternating steps) than during air stepping (~3%), suggesting that rhythmic ground contact may promote an alternating gait (Bradley and Smith, 1988b). However, in recent experiments in intact neonatal rats, in which a substrate (elastic, stiff, or none) was placed beneath their limbs so that the feet could make plantar surface contact with the substrate, pups treated with quipazine showed significantly more alternating fore- and hindlimb steps than pups treated with saline (Brumley et al., 2012). In rats with a neonatal spinal cord transection, the fictive locomotor pattern induced by excitatory amino acids does not exhibit any left/right alternation. However, strong alternation is restored when 5-HT is added to the bath (Norreel et al., 2003).

Serotonergic 5-HT_2_ and 5-HT_7_ receptor agonists have repeatedly been shown to promote locomotor recovery after SCI in adult rodents (Barbeau and Rossignol, 1990; Antri et al., 2002; Kao et al., 2006; Landry et al., 2006; Ung et al., 2008; Courtine et al., 2009; Murray et al., 2010; Jordan and Slawinska, 2011; Musienko et al., 2011; van den Brand et al., 2012). Combined activation of both receptor subtypes is more effective than activation of either receptor alone (Antri et al., 2005; Landry et al., 2006; Courtine et al., 2009; Sławińska et al., 2012). In rats with a complete thoracic spinal cord transection, grafts of embryonic serotonergic neurons improve locomotor recovery (Feraboli-Lohnherr et al., 1997; Ribotta et al., 2000; Majczyński et al., 2005; Sławińska et al., 2013). Importantly, both inter- and intralimb coordinations are improved by grafting embryonic 5-HT neurons after SCI in adult rats and the effectiveness of the transplants arises from intrinsic activation of 5-HT_2_ and 5-HT_7_ receptors (Sławińska et al., 2000, 2013).

## Modulation of inhibitory synaptic transmission by 5-HT_2_ and 5-HT_7_ receptors

There are, in principle, various ways through which 5-HT may strengthen inhibitory synaptic transmission. The most obvious explanation for the improvement of inter- and intralimb alternating motor activity following activation of 5-HT_2_ and 5-HT_7_ receptors is that this activation excites inhibitory interneurons responsible for coordinating flexor/extensor and left/right activity (Aggelopoulos et al., 1996; Pearlstein et al., 2005; Sławińska et al., 2013). Exogenously applied 5-HT (Lewis et al., 1993; Shen and Andrade, 1998; Abi-Saab et al., 1999; Xie et al., 2012) and endogenous 5-HT (Iwasaki et al., 2013) have been shown to activate GABAergic and/or glycinergic interneurons *via* 5-HT_2_ receptors in the CNS including the spinal cord. Activation of 5-HT_2A/2C_ receptors enhances glycine and/or GABA responses in spinal neurons in the rat (Xu et al., 1996, 1998; Li et al., 2000) and spontaneous inhibitory postsynaptic currents in the substantia gelatinosa (Xie et al., 2012). These effects involve, at least in part, a presynaptic facilitation of GABA/glycine release (Wang and Zucker, 1998; Xie et al., 2012). It has been shown that 5-HT_7_ receptor activation in the hippocampal CA1 area results in an enhancement of GABAergic transmission *via* two mechanisms (Tokarski et al., 2011). The first one involves an enhancement of excitatory glutamatergic input to GABAergic interneurons and is likely to be mediated by presynaptic 5-HT_7_ receptors. The second effect, most likely related to the activation of 5-HT_7_ receptors located on interneurons, results in an enhancement of GABA release.

Developmental studies provide interesting information about 5-HT modulation of inhibitory synaptic transmission. Maturation of inhibition in the lumbar spinal cord occurs during perinatal development in rodents. The key events are as follows: (1) the density of glycine currents (Gao and Ziskind-Conhaim, 1995) and receptors (Sadlaoud et al., 2010) increases whereas that of GABA_A_ currents and receptors drop concomitantly. (2) Inhibitory postsynaptic potentials switch from depolarizing to hyperpolarizing (Takahashi, 1984; Wu et al., 1992; Gao et al., 1998a; Jean-Xavier et al., 2006; Delpy et al., 2008; Stil et al., 2011), mostly due to the up-regulation of KCC2 expression (Jean-Xavier et al., 2006; Stil et al., 2009). A neonatal spinal cord transection at birth, which removes all descending modulatory influences from the brainstem, prevents both the depolarization-to-hyperpolarization switch (Jean-Xavier et al., 2006; Bos et al., 2013) and the developmental down-regulation of GABA_A_ currents and receptors (Sadlaoud et al., 2010). Interestingly, up-regulation of glycine receptors is not affected by spinal transection. Chronic treatment with the 5-HT_2_ receptor agonist, DOI, throughout the first postnatal week restores the hyperpolarizing shift of the chloride equilibrium potential (Bos et al., 2013) and the down-regulation of GABA_A_ receptors, without any significant effect on glycine receptors (Sadlaoud et al., 2010). These data suggested that 5-HT plays a role in the maturation of GABAergic synaptic transmission but that the up-regulation of glycinergic receptors does not depend on descending modulation from the brainstem.

Because the strength of inhibition depends on [Cl^−^]_i_, 5-HT, in principle, may strengthen inhibitory synaptic transmission by increasing KCC2 function. This hypothesis is supported by recent results showing that activation of 5-HT_2A_ receptors shifts the chloride equilibrium potential in the hyperpolarizing direction (Bos et al., 2013). This effect is mediated by an up-regulation of KCC2 function and involves a protein kinase C (PKC)-dependent mechanism. After SCI, acute addition of a specific 5-HT_2A_ receptor agonist, TCB-2, restores endogenous inhibition and thereby reduces spasticity and restores left-right alternation during fictive locomotion (Figures 1C,D; Bos et al., 2013). Interestingly, 5-HT_2A_ and 5-HT_2B/2C_ receptors were shown in the latter study to have opposite effects on KCC2 function. Consistent with these observations, 5-HT_2A_ and 5-HT_2C_ receptors exert opposing effects on both locomotor activity in mice and spinal reflexes in rats (Machacek et al., 2001; Halberstadt et al., 2009). As 5-HT_2B/2C_ receptors become constitutively active (spontaneously active without 5-HT) after SCI (Murray et al., 2010, 2011), this constitutive activity may be partly responsible for the depolarizing shift of the chloride equilibrium potential after SCI (Boulenguez et al., 2010).

## Conclusions and future directions

This review has shown that 5-HT plays a critical role in shaping the locomotor pattern by promoting left-right and flexor-extensor alternation, thereby raising the question of whether serotonin descending systems should be formally included as components of the CPGs for locomotion (Jordan and Slawinska, 2011). According to the initial definition of CPGs (Grillner and Wallén, 1985), “the term CPGs refers to function, not a circumscribed anatomical entity. The individual neurons that constitute the CPG may in principle be located in widely separate parts of the central nervous system”.

As combined exogenous application of both 5-HT_7_ and 5-HT_2_ receptor agonists is more effective than activation of either of these receptors alone (Antri et al., 2005; Landry et al., 2006; Musienko et al., 2011; Sławińska et al., 2012), it will be important to identify whether the mechanisms by which 5-HT_7_ receptors affect alternating motor activities also involve chloride homeostasis as is the case for 5-HT_2A_ receptors. Although the present review focused on serotonin, SCI removes not only serotonergic inputs but also dopaminergic and noradrenergic inputs to neurons below the lesion. The contribution of these pathways to the alternating locomotor pattern and regulation of chloride homeostasis should be investigated further.

## Conflict of interest statement

The authors declare that the research was conducted in the absence of any commercial or financial relationships that could be construed as a potential conflict of interest.
